# Biomechanical Analysis of Latin Dancers’ Lower Limb during Normal Walking

**DOI:** 10.3390/bioengineering10101128

**Published:** 2023-09-25

**Authors:** Xiangli Gao, Datao Xu, Fengfeng Li, Julien S. Baker, Jiao Li, Yaodong Gu

**Affiliations:** 1 Faculty of Sports Science, Ningbo University, Ningbo 315211, China; gaoxiangli1@aliyun.com (X.G.); xudatao3@gmail.com (D.X.); lifengfeng1999@hotmail.com (F.L.); 2 Faculty of Engineering, University of Pannonia, 8201 Veszprem, Hungary; 3 Department of Sport and Physical Education, Hong Kong Baptist University, Hong Kong 999077, China; 4 Faculty of Engineering, University of Szeged, 6724 Szeged, Hungary

**Keywords:** walking, Latin dancer, lower limb biomechanics, gait pattern

## Abstract

Latin dance involves fundamental walking steps, integral to the dance process. While resembling daily walking, Latin dance demands higher balance levels, necessitating body adjustments by dancers. These adaptations affect dancers’ gait biomechanics, prompting our study on gait differences between Latin dancers (LDs) and non-dancers (NDs). We enlisted 21 female Latin dancers and 21 subjects based on specific criteria. Participants executed walking tasks, with an independent sample *t*-test for 1-dimensional statistical parameter mapping (SPM 1d) analyzing stance phase variations between LDs and NDs. Notably, significant differences in ankle and hip external rotation were evident during the 16.43–29.47% (*p* = 0.015) and 86.35–100% (*p* = 0.014) stance phase. Moreover, pronounced distinctions in rectus Achilles tendon force (ATF) (12.83–13.10%, *p* = 0.049; 15.89–80.19%, *p* < 0.001) and Patellofemoral joint contact force (PTF) (15.85–18.31%, *p* = 0.039; 21.14–24.71%, *p* = 0.030) during stance were noted between LDs (Latin dancers) and NDs (Non-dancers). The study revealed dancers’ enhanced balance attributed to external ankle rotation for dance stability, coupled with augmented Achilles tendon and patellofemoral joint strength from prolonged practice. Moreover, integrating suitable Latin dance into rehabilitation may benefit those with internal rotation gait issues.

## 1. Introduction

With the evolution of Latin dance, an increasing number of scholars have taken interest in its significance. Research has revealed that Latin dance influences dancers’ gait patterns and enhances their balance [1,2,3]. The achievement of good balance in dancers relies on the coordination of the somatosensory, visual, and vestibular systems [4,5]. When dancers modify their movements, their body weight sways between their feet, prompting the need to maintain balance. As a result, dancers alternate between balancing on one foot and both feet. However, it is more advantageous for dancers to shift their weight onto one foot, facilitating seamless movement transitions. The diverse methods employed to maintain body balance patterns can produce varying effects on the biomechanics of the lower limbs. Optimal balance is crucial for dancers to effectively showcase their movements, granting them increased confidence. Conversely, an imbalance can make dancers susceptible to joint injuries. Notably, the ankle joint plays a vital role in maintaining balance, given its connection to the lower extremities in contact with the ground. Ankle sprains rank among the most common injuries in sports [6,7,8]. Moreover, it is important to recognize that ankle sprains tend to recur, potentially leading to chronic ankle instability [9,10]. The balance of dancers is intricately linked to the biomechanics of their lower extremities, with the joints and muscles in these regions assuming a pivotal role in maintaining stability.

With the remarkable advancements in biomechanics, a multitude of studies have explored the biomechanical gait of dancers, leading to intriguing observations regarding the profound impact that dance has on individuals’ balance [11,12,13]. Notably, biomechanics research has focused extensively on gait disorders among the elderly, and it has been suggested that individuals aged 65 and above face a significant 30 to 60 percent likelihood of experiencing balance loss and falls. To investigate this, researchers conducted a meticulous comparison of muscle strength tests and postural balance assessments between older LDs and older NDs, ultimately revealing the older LD group’s commendable superiority in performance [2,14]. Furthermore, although limited in scope, research has provided compelling evidence that LDs showcase enhanced balance and gait performance when compared to NDs [1,2,3,15]. The assessment of balance of the subjects involved the collection of kinematic data through one-legged stance tests, where a smaller range of motion during the test indicated better balance (necessitating less movement to maintain balance) [1]. Moreover, dynamic data derived from measuring dancers’ muscle strength plays a crucial role in assessing their balance. To accomplish this, the article employed a comprehensive battery of five tests and subsequently compared the obtained results through the utilization of computerized dynamic postural imaging [3]. The parameters associated with the center of pressure (COP) play a pivotal role in evaluating human gait stability [16]. Previous research has indicated that a 15-week intervention involving physical dance training for the elderly yielded noteworthy outcomes: A reduction in the COP area as well as the COP distance. It can be observed that Latin dance training has an impact on the human body’s COP. Therefore, we predict significant differences in COP between LDs and NDs, with LDs exhibiting a smaller range of COP activity compared to NDs.

In Latin dance, dancers perform actions such as flexion, extension, and rotation with their lower limbs. Engaging in Latin dance for an extended period can potentially strengthen the Achilles tendon. The Achilles tendon is a crucial part of the lower limb that effectively stores and absorbs energy during movement [17,18,19]. Increased Achilles tendons force (ATF) also contributes to improving ankle joint stability [20]. This is because the Achilles tendon is a vital structure connecting the heel and lower leg muscles [18]. When the Achilles tendon becomes stronger, it can better support the body’s weight and the forces generated during movement. During activities like exercise and sports, the lower limbs experience significant impact and pressure [21]. If the Achilles tendon lacks strength, it may not effectively transmit and absorb these forces, leading to instability in the lower limbs and a higher risk of sports injuries [20]. The knee joint plays a crucial role in maintaining body balance [22,23], and the patellofemoral joint contact force (PTF) is a contributing factor to knee joint stability [24]. Acting as a mechanical pulley, the patella enhances the lever arm of the quadriceps tendon, thereby improving the efficiency of the quadriceps muscle during knee extension [25]. Additionally, it aids in preventing lateral dislocation of the patella and provides stability during knee flexion and extension movements. Moreover, an increase in PTF can enhance knee joint stability to some extent. The PTF occurs between the femur and the tibia and constitutes one of the components of the knee joint. A rise in the contact force at the patellofemoral joint indicates a larger contact area or a more even distribution of forces between the bones. This can help alleviate pressure on specific areas, thereby improving knee joint stability to a certain degree. Based on previous research, we can calculate the ATF and PTF of participants. 

Gait reflects numerous indicators of the body’s functioning [26,27,28]. The Latin dance walk is very similar to walking in daily life. Walking serves as one of the essential basic steps in Latin dance, encompassing the entire dance process. A notable difference is that dancers need to lead their bodies forward using their toes. However, this poses a significant challenge for dancers in maintaining their balance, particularly for novices. Throughout the extensive dance careers of dancers, maintaining stability in balance leads to biomechanical changes in the lower extremities. Therefore, this study compares long-term LDs with NDs by calculating specific metrics, offering fresh insights for individuals dealing with gait disorders. To accomplish this, we collected joint kinematics and dynamics data from the subjects, utilizing cameras and force plates. This comprehensive approach enabled us to examine the intricate biomechanical variances in the lower extremities between LDs and NDs, providing information on the unique characteristics and potential advantages of LDs in this domain.

## 2. Materials and Methods

### 2.1. Participants

Sample sizes were calculated using G-Power software (version: 3.1.9.7; Henry University of Düsseldorf, Düsseldorf, Germany). A power analysis of variance was performed for independent sample t-tests with an effect size of 0.8 (significance level: 0.05 power: 0.8), and a total of 21 professional female LDs were recruited for the study (height: 163.29 ± 3.61 cm; age: 23.33 ± 1.05 years; body weight: 51.86 ± 1.55 kg). Each had a minimum of five years of dance experience, with at least 2–3 training sessions per week, with each session lasting no less than 45 min. The control group recruited 21 general female subjects (height: 164.2 ± 3.31 cm; age: 21.33 ± 1.33 years; weight: 51.3 ± 1.89) without any foundation in dance. The participants did not have any sports injuries for a year prior to the study. All participants were informed of the procedures, conditions, and requirements of the study and provided written informed consent prior to data collection. The ethics review committee of Ningbo University approved the study. This approval was obtained prior to participation in the study (Protocol Code: RAGH20220618).

### 2.2. Experimental Protocol and Equipment

**Figure 1:**
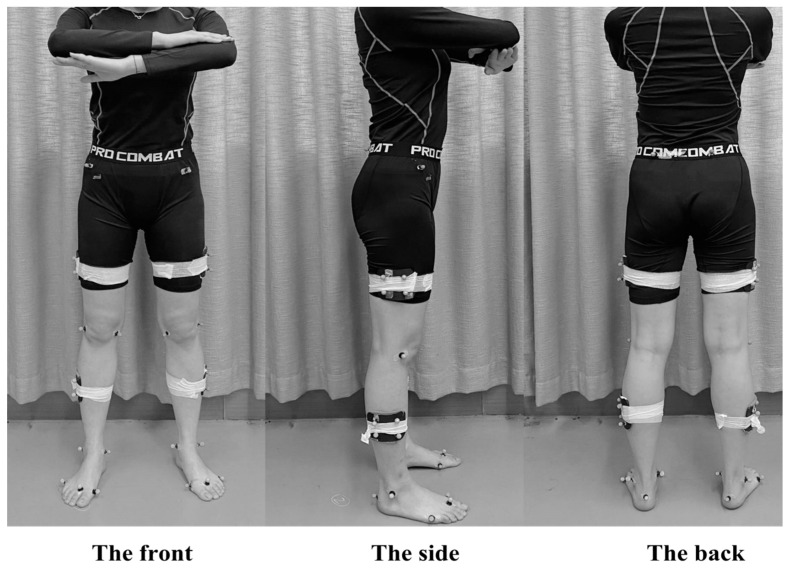
Illustration of the subject’s reflection markers.

The sports biomechanics laboratory of Ningbo University served as the site for all the tests used in this study. A total of 36 standard reflex points (diameter: 12.5 mm) were affixed to the subject’s lower limbs and pelvis to capture the subject’s movement [29,30]. The marker points’ precise locations are depicted in Figure 1. The locations of the markers included the first and fifth metatarsal bones, medial and lateral knee joints, medial and lateral ankle joints, lateral heel, distal joint of the second digit phalangeal bone, anterior and posterior sides of the left and right iliac bone, medial side of the outer thigh and lower leg, and distal joint of the first and second digits of the phalangeal bone. Eight infrared cameras were used to record the data using the Vicon motion capture system in conjunction with a ground force plate used to collect kinetic data (AMTI, Watertown, MA, USA). The sampling frequencies for kinematics and kinetic were 200 and 1000 Hz, respectively. Vicon Nexus software was used to collect kinematic and dynamic data simultaneously [31].

### 2.3. Experimental Procedure

Before the commencement of the experiment, all participants were instructed to coil their hair, wear tights, and perform the experiment barefoot to ensure consistency and eliminate potential confounding variables. The experiment was divided into three distinct parts to systematically assess various aspects of the participants’ lower extremity biomechanics. (1) The participants engaged in a 10 min warm-up session, which involved jogging at a speed of 8 km/h. This warm-up not only prepared the participants physically but also allowed them to acclimatize and familiarize themselves with the experimental environment and become familiar with the experimental procedures. (2) The formal experiment involved the collection of static data. Participants were positioned in an anatomical posture and asked to stand on the force plate. This static data acquisition provided a baseline reference for subsequent analyses and comparisons. (3) Following the collection of static data, the dynamic data collection phase commenced. Participants were given a command and instructed to walk five meters in a straight line from one side of the force plate to the other at their own comfortable pace. This allowed for the assessment of their dynamic lower limb movements during walking. Throughout the experiment, an inspector was assigned to closely monitor each participant. In any instance where a participant’s foot was not placed properly on the force plate or if their gait deviated significantly, the measurement was considered invalid and required re-measurement to maintain data accuracy and integrity. To ensure the test results were not compromised, speed meters were installed on either side of the participants’ direction of motion. The walking speed was pre-set within the range of 1.05–1.43 m/s before each participant’s experiment [32,33]. If a participant’s walking speed exceeded the specified range, the corresponding data were deemed invalid and excluded from the analysis to maintain the consistency and reliability of the results.

### 2.4. Data Collection and Processing

According to previous studies, the initial contact point is defined as the vertical reaction force exceeding 10 N after the right foot makes contact with the ground [34,35]. The kinematic and kinetics data were collected using Vicon Nexus and subsequently imported into Visual-3D software provided by C-Motion, Inc. (Germantown, MD, USA) for modeling and further processing. To determine the optimal signal-to-noise ratio of the collected data, a subset residual analysis was conducted, and the data were filtered at 10 and 20 Hz. We collected data from participants using a three-dimensional force plate and extracted the vertical, anterior-posterior, and medial-lateral ground reaction forces (GRF). Subsequently, we derived the COP excursion for participants, encompassing both the anterior-posterior range of COP movement and the medial-lateral range of COP movement [36].

Based on previous research, Achilles Tendon Force (ATF) was determined by dividing the ankle flexion moment found in the C3D file obtained from Vicon by the moment arm. The ankle flexion angle has been denoted as α in earlier studies. A previous investigation utilized Magnetic Resonance Imaging (MRI) to ascertain the moment arm $M_{arm},$ which was computed using the following formula [37,38]:

$$M_{arm}=-0.5910+0.08297\theta_{\alpha }-0.0002606\theta_{\alpha }^{2}$$

$M_{a}$ represents the plantar flexion moment of the ankle joint, and ATF is calculated using the following formula [39]:

$$ATF=\frac{M_{a}}{M_{arm}}$$

The patellofemoral joint contact force (PTF) was estimated as a function of the knee flexion angle (x) and extensor moment (Mk) [40]. Calculation of the flexion knee angle based on the nonlinear equation of the quadriceps arm is as follows [41]:

$$Lq=0:00008x^{3}-0:013x^{2}+0:28x+0:046$$

Quadriceps strength (Fq) is calculated by the following formula:

Fq=Mk/Lq

The constant k of the angular position (x) of the knee joint is calculated using the non-linear equation described in [42]:

$$K=\frac{\left(0.462+0.00147x^{2}-0.0000384x^{2}\right)}{\left(1-0.0162x^{2}+0.000155x^{2}-0.000000698x^{3}\right)}$$

The PTF was calculated using quadriceps muscle force (Fq) and constant (k):

PTF=Fq∗k

### 2.5. Statistical Analysis

The resulting data were then imported into MATLAB R2022a (MathWorks, MA, USA) for further processing. For non-parametric data, the Wilcoxon-matched signature rank test was employed. The dynamics and kinematics data were analyzed using MATLAB, utilizing statistical parameter mapping for the analysis [43,44]. Custom MATLAB scripts were used to extract all-time series curves, extending them to 101 data points, representing the 0–100% landing phase (with a significance threshold of 0.05). SPSS 27.0 (Chicago, IL, USA) for Windows software was utilized to analyze the data. Independent samples t-tests assessed significant differences in peak variables between the LDs and NDs, with a significance threshold set at 0.05 (*p* < 0.05). Finally, the resulting data were inputted into Origin 2021 software for visualization and plotting.

## 3. Results

### 3.1. Results of the Kinetics and Kinematics in the Sagittal Plane

**Figure 2:**
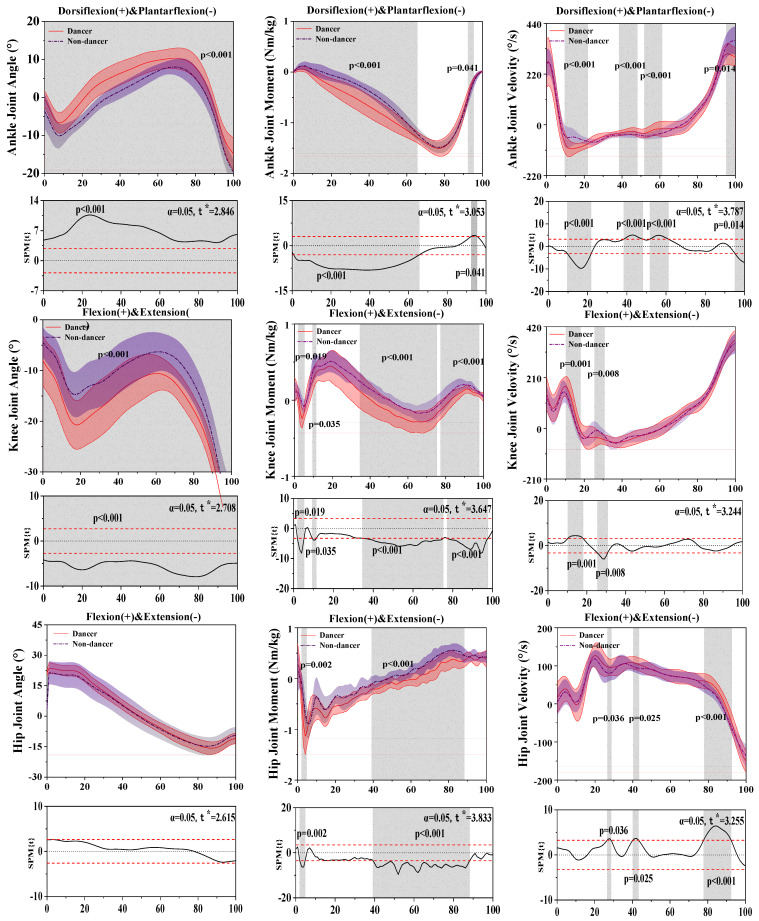
Illustration of the results between LD and ND lower limbs showing the statistical parametric mapping outputs for the angle, moment, and velocity of the ankle, knee, and hip during the stance phase. The values of t* are shown on the left of each image. Grey shades represent the significant differences and t-values of the SPM or all participants, and dashed red lines represent the results at *p* = 0.05.

Figure 2 shows the difference in kinematics and dynamics between LDs and NDs in the sagittal plane.

For the results of the ankle joint, SPM analysis revealed that LDs depicted a significantly greater plantarflexion angle than NDs during the 0–100% (*p* < 0.001) stance phase, a significantly greater plantarflexion moment than NDs during the 0.71–65.59% (*p* < 0.001) and 92.35–95.40% (*p* = 0.041) stance phases, and a significantly greater plantarflexion velocity than NDs during the 9.65–21.81% (*p* < 0.001), 38.40–48.15% (*p* < 0.001), 51.75–61.24% (*p* < 0.001), and 95.14–100% (*p* = 0.014) stance phases. 

For the results of the knee joint, SPM analysis revealed that LDs depicted a significantly greater extension angle than NDs during the 1.91–5.24% (*p* = 0.019) stance phase. There were significant differences in the knee moment between LDs and NDs during the 1.91–5.24% (*p* = 0.019), 9.40–11.40% (*p* = 0.035), 34.57–75.27% (*p* < 0.001), and 77.21–97.70% (*p* < 0.001) stance phases. There were significant differences in the velocity between LDs and NDs during the 10.23–18.06% (*p* = 0.001) and 25.43–30.72% (*p* = 0.008) stance phases.

For the results of the hip joint, there were significant differences in the hip moment between LDs and NDs during the 2.04–4.73% (*p* = 0.002) and 39.18–88.22% (*p* < 0.001) stance phases and a significantly greater flexion velocity than NDs during the 26.59–28.65% (*p* = 0.036), 40.25–43.29% (*p* = 0.025), and 77.81–92.40% (*p* < 0.001) stance phases.

Table 1 displays that dorsiflexion of the angle of the ankle (*p* < 0.001) and flexion of the knee (*p* = 0.003) had significant differences between LDs and NDs during the stance phase. Additionally, flexion (*p* = 0.006) and extension (*p* < 0.001) of knee moment had significant differences between LDs and NDs during the stance phase.

### 3.2. Results of the Kinetics and Kinematics in the Coronal Plane

**Figure 3:**
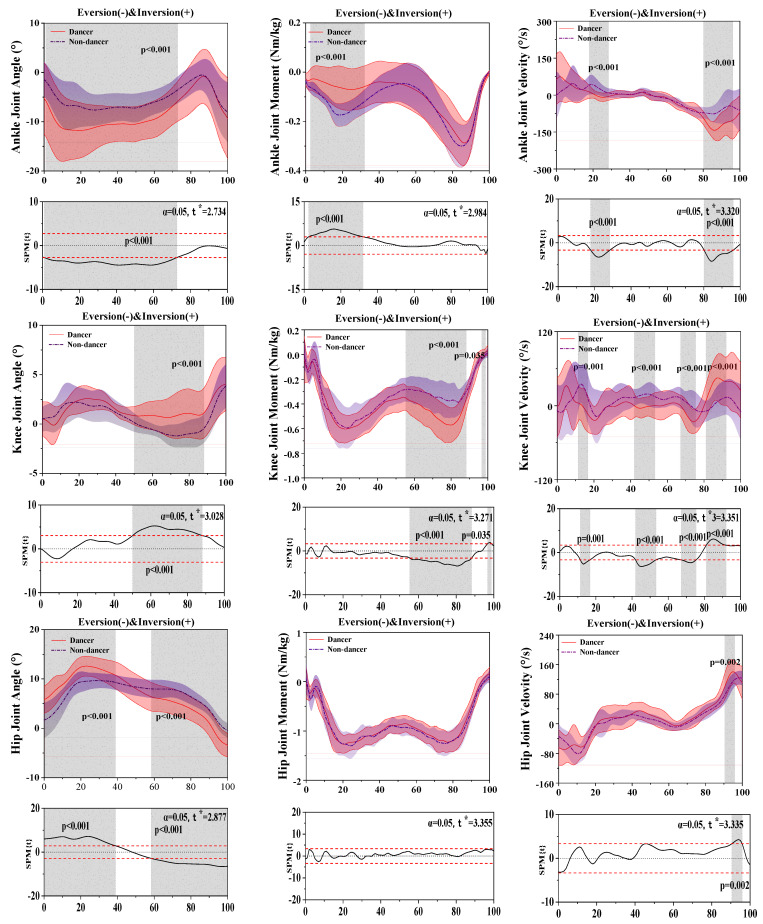
Illustration of the results between LDs and NDs lower limbs showing the statistical parametric mapping outputs for the angle, moment, and velocity of the ankle, knee, and hip during the stance phase. The values of t* are shown on the left of each image. Grey shades represent the significant differences and t-values of the SPM for all participants, and dashed red lines represent the results at *p* = 0.05.

Figure 3 shows the difference in kinematics and dynamics between LDs and NDs in the coronal plane.

For the results of the ankle joint, SPM analysis revealed that LDs depicted a significantly greater eversion angle than NDs during the 0.88–72.71% (*p* < 0.001) stance phase, a significantly smaller moment than NDs during the 2.44–32.09% (*p* < 0.001) stance phase, and significant differences in the velocity between LDs and NDs during the 17.81–28.43% (*p* < 0.001) and 80.13–96.17% (*p* < 0.001) stance phases.

For the results of the knee joint, SPM analysis revealed that LDs depicted a significantly greater abduction angle than NDs during the 50.08–88.03% (*p* = 0.019) stance phase, a significantly greater moment than NDs during the 55.36–88.23% (*p* < 0.001) and 96.72–98.76% (*p* = 0.035) stance phases, and a significantly greater velocity than NDs during the 11.36–16.73% (*p* = 0.001), 41.87–53.26% (*p* < 0.001), 67.16–75.47% (*p* < 0.001), and 81.06–92.12% (*p* < 0.001) stance phases.

For the results of the hip joint, SPM analysis revealed that LDs depicted a significantly greater abduction angle than NDs during the 0–38.92% (*p* = 0.019) stance phase and a significantly smaller adduction angle than NDs during the 58.37–100% (*p* < 0.001) stance phase.

Table 2 displays that eversion of the angle of the ankle (*p* = 0.016), adduction of the knee (*p* = 0.014), and abduction (*p* < 0.001) and adduction of the hip (*p* < 0.001) had significant differences between LDs and NDs during the stance phase. 

### 3.3. Results of the Kinetics and Kinematics in the Horizontal Plane

**Figure 4:**
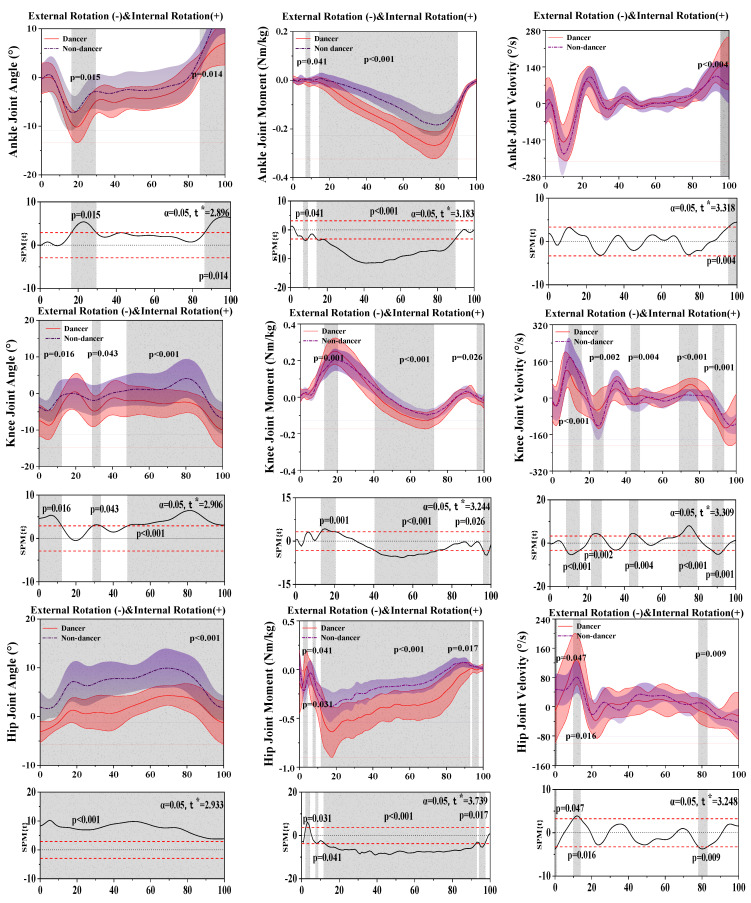
Illustration of the results between LDs and NDs lower limbs showing the statistical parametric mapping outputs for the angle, moment, and velocity of the ankle, knee, and hip during the stance phase. The values of t* are shown on the left of each image. Grey shades represent the significant differences and t-values of the SPM for all participants, and dashed red lines represent the results at *p* = 0.05.

Figure 4 shows the difference in kinematics and dynamics between LDs and NDs in the horizontal plane.

For the results of the ankle joint, SPM analysis revealed that LDs depicted a significantly greater external rotation angle than NDs during the 16.43–29.47% (*p* = 0.015) and 86.35–100% (*p* = 0.014) stance phase and a significantly greater moment than NDs during the 6.94–9.32% (*p* = 0.041) and 14.30–89.68% (*p* < 0.001) stance phases.

For the results of the knee joint, SPM analysis revealed that LDs depicted a significantly greater external rotation angle than NDs during the 0–12.28% (*p* = 0.016), 28.94–33.39% (*p* = 0.043), and 47.72–100% (*p* < 0.001) stance phases, a significantly greater moment than NDs during the 13.03–20.41% (*p* = 0.001) and 40.51–72.66% (*p* < 0.001) stance phases, and significant differences in the velocity between LDs and NDs during the 8.77–15.86% (*p* < 0.001), 22.21–27.72% (*p* = 0.002), 42.65–47.45% (*p* = 0.004), 68.95–79.20% (*p* < 0.001), and 87.11–93.38% (*p* = 0.001) stance phases.

For the results of the hip joint, SPM analysis revealed that LDs depicted a significantly greater external rotation angle than NDs during the 0–100% (*p* < 0.001) stance phase, a significantly greater moment than NDs during the 2.09–4.36% (*p* = 0.031), 7.30–8.74% (*p* = 0.041), 11.73–92.84% (*p* < 0.001), and 94.08–97.46% (*p* = 0.017) stance phases, and a significantly greater velocity than NDs during the 9.98–13.87% (*p* = 0.016) and 78.03–82.90% (*p* = 0.009) stance phase.

Table 3 displays that external rotation (*p* = 0.001) and internal rotation (*p* < 0.001) of the angle of the ankle and external rotation of the moment of the ankle (*p* < 0.001) had significant differences between LDs and NDs during the stance phase. Additionally, external rotation (*p* = 0.004) and internal rotation (*p* < 0.001) of the knee moment had significant differences between LDs and NDs during the stance phase.

### 3.4. Results of the Ground Reaction Force, Patellofemoral Joint Contact Force, and Achilles Tendon Force

**Figure 5:**
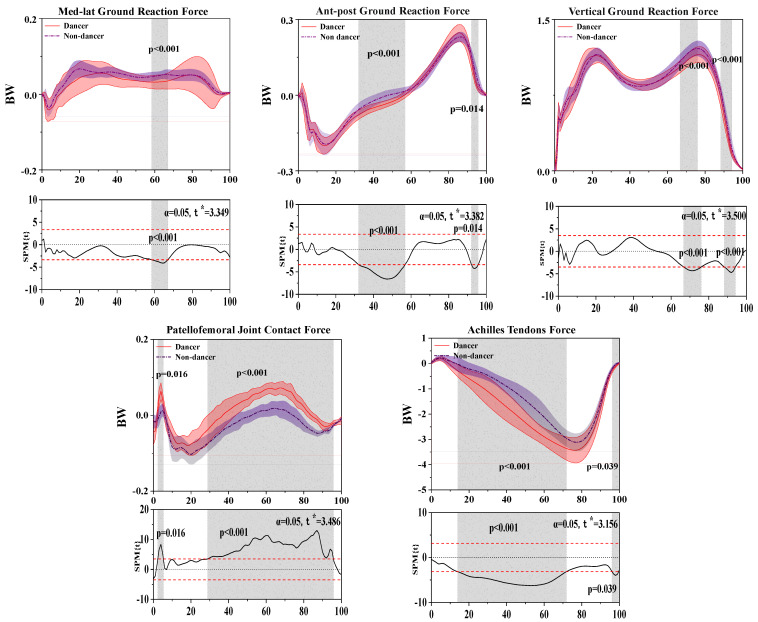
Illustration of the results between LDs and NDs lower limbs showing the statistical parametric mapping outputs for the ground reaction force Achilles tendon force and Patellofemoral joint contact force during the stance phase. The values of t* are shown on the left of each image. Grey shades represent the significant differences and t-values of the SPM for all participants, and dashed red lines represent the results at *p* = 0.05.

Figure 5 shows the difference in ground reaction force, Achilles tendon force, and Patellofemoral joint contact force between LDs and NDs.

In the sagittal plane, SPM analysis revealed that LDs depicted a significantly smaller force than NDs during the 58.36–67.02% (*p* < 0.001) stance phase.

In the coronal plane, SPM analysis revealed that LDs depicted a significantly greater force than NDs during the 32.02–56.81% (*p* < 0.001) stance phase.

In the horizontal plane, SPM analysis revealed that LDs depicted a significantly smaller force than NDs during the 6.67–75.98% (*p*< 0.001) and 88.25–94.18% (*p* < 0.001) stance phases.

For the results of patellofemoral joint contact force, SPM analysis revealed that LDs depicted a significantly greater force than NDs during the 2.37–5.34% (*p* = 0.016) and 28.78–95.78% (*p* < 0.001) stance phases.

For the results of Achilles tendon force, SPM analysis revealed that LDs depicted a significantly greater force than NDs during the 13.97–71.85% (*p* < 0.001) and 96.10–99.60% (*p* = 0.039) stance phases.

### 3.5. Results of the COP (Center of Pressure) Trajectory

**Figure 6:**
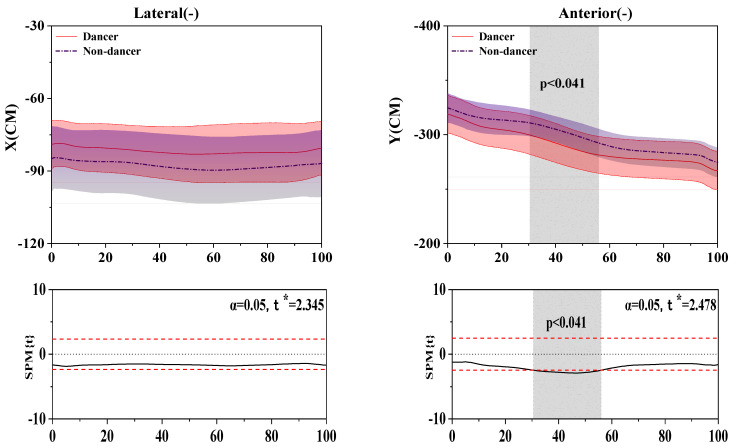
Illustration of the results between LDs and NDs lower limbs showing the statistical parametric mapping outputs for ML-COP and AP-COP during the stance phase. The values of t* are shown on the left of each image. Grey shades represent the significant differences and t-values of the SPM for all participants, and dashed red lines represent the results at *p* = 0.05.

Figure 6 shows the difference in COP between LDs and NDs.

For the results of AP-COP, SPM analysis revealed that LDs depicted a significantly smaller force than NDs during the 30.47–55.94% (*p* = 0.041) stance phase.

## 4. Discussion

This comprehensive study investigated the intricate biomechanical distinctions of the lower extremities between LDs and NDs during the gait process. By meticulously analyzing the research outcomes, a multitude of significant differences in gait patterns between LDs and NDs becomes apparent. By computing COP-related parameters, it was observed that there were no significant differences in ML-COP between LDs and NDs. However, during the 30.47–55.94% standing phase, NDs exhibited a larger fluctuation range in AP-COP compared to LDs, indicating a lesser degree of stability in NDs. The study’s primary hypothesis postulated that there exist substantial disparities in lower limb joints between LDs and NDs. The findings confirm this hypothesis by revealing a distinction in the foot rotation angle between the LDs and NDs. Notably, previous research has shown that individuals tend to exhibit external rotation of the ankle joint as a means to maintain stability when confronted with instability [45]. The intricate choreography was intrinsic to female Latin dancers, be it in solo performances or partnered routines, and necessitated an abundance of rotational movements. In Latin dance, dancers strive to maintain alignment between their front and back feet throughout their performances; nevertheless, young dancers who lack balance may encounter difficulties in achieving this alignment. As a result, they may rely on external rotations of the ankle to sustain balance and facilitate the execution of dance sequences. The long-term reliance of dancers on ankle joint external rotation to maintain stability may potentially affect their gait in daily life. This observation highlights the far-reaching implications of dance-related biomechanical adaptations on individuals’ overall movement mechanics beyond the dance floor.

Previous studies have consistently reported a significant increase at the moment when the foot undergoes external rotation, aligned with our research findings that highlight LDs’ significantly greater moment compared to NDs during walking. This observation suggests that LDs rely on an increased moment to accommodate the rotational demands of their footwork. Interestingly, in situations where stability necessitates foot rotation inwards and outwards, both the internal and external binding forces, as well as the internal and lateral rollover moments, exhibit a substantial increase within the knee joint [45]. Specifically, during the 13.03–20.41% (*p* = 0.001) and 40.51–72.66% (*p* < 0.001) stance phases, LDs demonstrated a greater knee moment compared to NDs, consequently leading to elevated knee compression and amplified knee impact force. These findings emphasize the vulnerability of the knee joint to potential injuries, as it serves as a critical component for attenuating and transferring the energy generated during movement. Such insights shed light on the intricate interplay between lower limb biomechanics, foot rotations, and the associated risk factors for knee-related injuries in dancers [46].

In addition to foot rotations, we analyzed the ATF in both LDs and NDs. Specifically, in the 13.97–71.85% (*p* < 0.001) and 96.10–99.60% (*p* < 0.039) stance phases, significant differences were observed, with LDs exhibiting a substantially greater ATF compared to NDs. This disparity may be attributed to the unique demands of Latin dance, where dancers strive to showcase optimal leg lines and visual effects by maximizing the extension of their insteps. Consequently, the ankle experiences frequent and pronounced movements, leading to enhanced muscular development around the ankle region and potentially increased strength in the Achilles tendon. Considering the significance of ATF in dancers’ balance, it is worth noting that previous studies have established a significant correlation between inadequate strength and an increased risk of falls [47,48,49,50,51]. Hence, the amplified ATF observed in LDs may serve as a vital contributing factor in maintaining balance and stability throughout their performances. These insights underscore the multifaceted interplay between lower limb biomechanics and the critical role of ATF in facilitating dancers’ equilibrium.

The knee joint, acting as a vital link between the ankle and the hip, assumes a crucial role in maintaining balance and stability. In line with our overarching hypothesis, we delved further into our analysis by calculating the PTF in both LDs and NDs. Notably, the patella, commonly referred to as the kneecap, influences the movement of the lower extremities by augmenting the quadriceps muscle [52]. Given the rigorous training and constant practice required in the field of Latin dance, LDs continually work on strengthening their legs. Drawing upon the insights garnered from our comprehensive SPM and SPSS analyses, we observed that LDs exhibited a significantly greater contact force in their patella joints compared to NDs. This heightened contact force could potentially contribute to enhanced muscle control in the knee joint, facilitating the dancers’ ability to maintain balance and stability during intricate dance moves. 

Consequently, ankle joint external rotation, ATF, and increased patellofemoral joint contact force are all potential factors contributing to the improvement of balance in dancers. We propose that when practicing Latin dance, dancers should conscientiously strive to maintain correct foot posture. Through dedicated and repetitive training, they can bolster the strength of their leg and ankle muscles, thereby fortifying overall stability while concurrently alleviating the strain exerted on the knees. This approach holds promise in reducing the likelihood of sports-related injuries and potentially prolonging dancers’ careers. By emphasizing the importance of proper foot alignment and comprehensive training, dancers can enhance both their performance quality and long-term physical well-being. While our study focused exclusively on female Latin dancers, we acknowledge the integral role played by male dancers in the realm of Latin dance. To comprehensively explore the nuances of gait differences between genders in Latin dance, future research endeavors should encompass male Latin dancers as well. By expanding the scope of our investigations to include male dancers, we can gain deeper insights into the interplay between gender and gait mechanics within the Latin dance domain, paving the way for more inclusive and nuanced studies in the future.

## 5. Conclusions

This study found that LDs have better balance than NDs, and the dancers’ better balance may be due to their use of the external rotation of the ankle to maintain balance during the dance and to the fact that their Achilles tendon strength and patellofemoral joint contact force have been enhanced by long-term dance practice. However, it is important to note that if dancers rely too heavily on external rotation of the ankle joint to maintain stability, this may increase the risk of potential injury. Therefore, we recommend that dancers keep their feet in the correct position when practicing the movements, which can be achieved by strengthening the leg muscles to improve stability. In addition, Latin dance can be used as a corrective method for those with ankle internal rotation gait disorders.

## Figures and Tables

**Table 1:** Comparison of sagittal plane joint angles, joint moments, and joint velocities (means ± standard) between LDs and NDs during the stance phase.

Joint	Parameters	Peak Value	Dancer Mean ± SD	Non-Dancer Mean ± SD	*p*-Value
Ankle	Angle (°)	Dorsiflexion	−14.89 ± 4.18	−19.65 ± 3.33	<0.001 *
		Plantarflexion	10.99 ± 2.58	8.44 ± 1.92	<0.001 *
	Moment (Nm/kg)	Dorsiflexion	−1.51 ± 0.16	−1.50 ± 0.12	0.63
		Plantarflexion	0.078 ± 0.042	0.14 ± 0.08	<0.001 *
	Velocity (°/s)	Dorsiflexion	120.23 ± 19.67	−92.86 ± 21.37	<0.001 *
		Plantarflexion	350.05 ± 54.05	375.22 ± 55.16	0.024 *
Knee	Angle (°)	Flexion	24.22 ± 2.44	21.62 ± 5.73	0.003 *
		Extension	−15.81 ± 3.09	−14.84 ± 4.46	0.216
	Moment (Nm/kg)	Flexion	0.64 ± 0.16	0.73 ± 0.15	0.006 *
		Extension	−1.27 ± 0.29	−1.02 ± 0.21	<0.001 *
	Velocity (°/s)	Flexion	143.92 ± 25.41	134.31 ± 17.43	0.039 *
		Extension	−150.85 ± 28.60	−136.36 ± 29.03	0.016 *
Hip	Angle (°)	Flexion	−42.82 ± 3.96	−38.15 ± 5.60	<0.001 *
		Extension	−7.18 ± 3.97	−3.79 ± 2.67	<0.001 *
	Moment (Nm/kg)	Flexion	−0.37 ± 0.12	−0.23 ± 0.09	<0.001 *
		Extension	0.51 ± 0.17	0.59 ± 0.15	0.017 *
	Velocity (°/s)	Flexion	−85.65 ± 25.09	−75.74 ± 22.83	0.041 *
		Extension	365.32 ± 39.26	350.76 ± 40.57	0.026 *

Note: “*” indicates a significant difference between LDs and NDs in the stance phase (*p* < 0.05).

**Table 2:** Comparison of coronal plane joint angles, joint moments, and joint velocities (means ± standard) between LDs and NDs during the stance phase.

Joint	Parameters	Peak Value	Dancer Mean ± SD	Non-Dancer Mean ± SD	*p*-Value
Ankle	Angle (°)	Eversion	−14.13 ± 6.07	−11.06 ± 3.63	0.16 *
		Inversion	1.22 ± 4.90	1.19 ± 3.16	0.97
	Moment (Nm/kg)	Eversion	−0.29 ± 0.09	−0.30 ± 0.09	0.71
		Inversion	0.03 ± 0.03	0.02 ± 0.02	0.14 *
	Velocity (°/s)	Eversion	−170.49 ± 46.36	−135.36 ± 51.84	0.53
		Inversion	130.11 ± 69.49	102.06 ± 30.66	0.06
Knee	Angle (°)	Abduction	−1.64 ± 1.36	−1.75 ± 1.00	0.703
		Adduction	5.17 ± 1.49	4.03 ± 2.13	0.014 *
	Moment (Nm/kg)	Abduction	−0.68 ± 0.13	−0.64 ± 0.17	0.165
		Adduction	0.11 ± 0.09	0.10 ± 0.10	0.761
	Velocity (°)	Abduction	−61.76 ± 30.49	−67.97 ± 28.56	0.340
		Adduction	86.72 ± 26.61	72.83 ± 29.02	0.026 *
Hip	Angle (°)	Abduction	13.00 ± 2.08	10.27 ± 1.44	<0.001 *
		Adduction	−3.43 ± 2.41	−0.82 ± 1.92	<0.001 *
	Moment (Nm/kg)	Abduction	0.27 ± 0.13	0.24 ± 0.18	0.237
		Adduction	−1.42 ± 0.20	−1.45 ± 0.20	0.380
	Velocity (°)	Abduction	153.36 ± 31.50	128.50 ± 18.64	<0.001 *
		Adduction	−107.99 ± 21.65	−95.60 ± 27.90	0.034 *

Note: “*” indicates a significant difference between LDs and NDs in the stance phase (*p* < 0.05).

**Table 3:** Comparison of horizontal plane joint angles, joint moments, and joint velocities (means ± standard) between LDs and NDs during the stance phase.

Joint	Parameters	Peak Value	Dancer Mean ± SD	Non-Dancer Mean ± SD	*p*-Value
Ankle	Angle (°)	External rotation	−7.92 ± 3.43	−10.39 ± 3.19	0.001 *
		Internal rotation	13.62 ± 4.59	7.23 ± 4.34	<0.001 *
	Moment (Nm/kg)	External rotation	−0.28 ± 0.05	−0.19 ± 0.05	<0.001 *
		Internal rotation	0.02 ± 0.10	0.23 ± 0.14	0.003 *
	Velocity (°/s)	External rotation	−196.33 ± 66.33	−221.41 ± 67.81	0.065
		Internal rotation	190.81 ± 66.01	154.27 ± 44.27	0.002 *
Knee	Angle (°)	External rotation	−8.42 ± 3.18	−11.10 ± 4.45	0.004 *
		Internal rotation	5.69 ± 4.42	1.94 ± 3.78	<0.001 *
	Moment (Nm/kg)	External rotation	−0.14 ± 0.04	−0.11 ± 0.34	<0.001 *
		Internal rotation	0.26 ± 0.08	0.21 ± 0.06	<0.001 *
	Velocity (°/s)	External rotation	−173.73 ± 57.28	−168.32 ± 33.60	0.612
		Internal rotation	175.26 ± 54.77	215.69 ± 57.82	0.001 *
Hip	Angle (°)	External rotation	−0.63 ± 2.43	−4.81 ± 1.46	<0.001 *
		Internal rotation	11.77 ± 3.70	5.21 ± 2.28	<0.001 *
	Moment (Nm/kg)	External rotation	−0.71 ± 0.25	−0.45 ± 0.14	<0.001 *
		Internal rotation	0.18 ± 0.11	0.15 ± 0.09	0.155
	Velocity (°/s)	External rotation	160.27 ± 61.25	105.98 ± 31.50	<0.001 *
		Internal rotation	−107.65 ± 36.74	−68.44 ± 28.05	<0.001 *

Note: “*” indicates a significant difference between LDs and NDs in the stance phase (*p* < 0.05).

## Data Availability

The data that support the findings of this study are available upon reasonable request from the corresponding author. The data are not publicly available due to privacy or ethical restrictions.
